# Assessment of a Spatiotemporal Deep Learning Approach for Soil Moisture Prediction and Filling the Gaps in Between Soil Moisture Observations

**DOI:** 10.3389/frai.2021.636234

**Published:** 2021-03-04

**Authors:** Mohamed ElSaadani, Emad Habib, Ahmed M. Abdelhameed, Magdy Bayoumi

**Affiliations:** ^1^ Department of Civil Engineering and Louisiana Watershed Flood Center, University of Louisiana at Lafayatte, Lafayette, LA, United States; ^2^ Department of Electrical and Computer Engineering, University of Louisiana at Lafayatte, Lafayette, LA, United States

**Keywords:** deep learning, soil moisture, LSTM, convolutional neural network, Louisiana (United States)

## Abstract

Soil moisture (SM) plays a significant role in determining the probability of flooding in a given area. Currently, SM is most commonly modeled using physically-based numerical hydrologic models. Modeling the natural processes that take place in the soil is difficult and requires assumptions. Besides, hydrologic model runtime is highly impacted by the extent and resolution of the study domain. In this study, we propose a data-driven modeling approach using Deep Learning (DL) models. There are different types of DL algorithms that serve different purposes. For example, the Convolutional Neural Network (CNN) algorithm is well suited for capturing and learning spatial patterns, while the Long Short-Term Memory (LSTM) algorithm is designed to utilize time-series information and to learn from past observations. A DL algorithm that combines the capabilities of CNN and LSTM called ConvLSTM was recently developed. In this study, we investigate the applicability of the ConvLSTM algorithm in predicting SM in a study area located in south Louisiana in the United States. This study reveals that ConvLSTM significantly outperformed CNN in predicting SM. We tested the performance of ConvLSTM based models by using a combination of different sets of predictors and different LSTM sequence lengths. The study results show that ConvLSTM models can predict SM with a mean areal Root Mean Squared Error (RMSE) of 2.5% and mean areal correlation coefficients of 0.9 for our study area. ConvLSTM models can also provide predictions between discrete SM observations, making them potentially useful for applications such as filling observational gaps between satellite overpasses.

## Introduction

In this study, we explore the performance of spatial Deep Learning (DL) neural network models in the field of hydrologic prediction. Namely, we focus on modeling Soil Moisture (SM) as a key hydrologic variable. SM plays a fundamental role in the water and energy exchange between the soil and atmosphere. It has a significant effect on many hydrologic processes and applications such as drought and flood prediction, water availability for evapotranspiration by plants, and irrigation planning (Koster 2004; Narasimhan and Srinivasan 2005; Norbiato et al., 2008; Fang et al., 2017). Hydrologic variables such as SM are known to exhibit significant spatiotemporal variability due to their inter-dependence on past and current conditions of related environmental and hydrometeorological variables (e.g., incoming long and shortwave radiation, temperature, available surface, and subsurface runoff, and evapotranspiration). Currently, these processes are commonly simulated using sets of physically or statistically based models that require tradeoffs between accuracy, resolution, and computational efficiency. These tradeoffs are inevitable given the current state of knowledge (i.e., the current understanding of how these physical processes take place) and technology (i.e., the time and resources needed to simulate these processes). On the other hand, data-driven DL models have the potential to aid, or in some cases be an alternative to, hydrologic models. DL models do not require complex statistical or physically-based equations targeting a single variable, but rather perform their predictions solely based on learning from a set of predictors that are related to the variable of interest. Moreover, once the model learning process is complete and a DL model has been developed, it can be easily stored and used to process future data in a significantly short amount of time. Compared to some physically-based models, deep learning models do not require specific operating systems to operate in. DL models can easily integrate more data for additional learning and improving their quality in the future as more data are available.

Two of the most relevant DL neural network algorithms for hydrologic prediction are Convolutional Neural Networks (CNN; LeCun et al., 2015) and Long Short-Term Memory networks (LSTM; Hochreiter and Schmidhuber, 1997). CNNs are powerful in identifying spatial patterns and strongly respond to spatial correlations that are dominantly present in hydrologic variables. On the other hand, LSTM is a class of Recurrent Neural Networks (RNN) that utilize feedback connections; this allows the LSTM algorithm to not only process single data points, but process an entire sequence of data. Therefore, LSTM is well suited for time series applications. These powerful DL algorithms started drawing the attention of the hydrologic community in recent years. Studies such as Hu et al., (2018) and Kratzert et al., (2018) assessed the performance of LSTM based DL models in forecasting daily and hourly runoff, respectively. Fang et al., (2017) used LSTM to predict observations of the Soil Moisture Active Passive (SMAP) V3 satellite using atmospheric forcings in combination with simulated SM from the Noah hydrologic model. The output from their LSTM model predicted SMAP observations with low Root Mean Squared Error (RMSE) and high correlation (R). Pan et al., (2019) used CNN-based DL models to improve rainfall estimates from weather models. Their CNN models outperformed statistical downscaling approaches such as linear regression, nearest neighbor, and random forest.

Despite the suitability of CNN and LSTM in spatial and temporal applications respectively, their special capabilities have only recently been combined. Xingjian et al., (2015) developed a new algorithm that combines the capabilities of CNN and LSTM, called Convolutional LSTM (referred to as ConvLSTM for the remainder of this article). Their study applied the ConvLSTM algorithm in rainfall nowcasting and was able to effectively predict rainfall intensities and reproduce the spatiotemporal properties of short-term rainfall fields. Moreover, ConvLSTM outperformed other models such as the Fully Connected LSTM (FC-LSTM; Srivastava et al., 2015) and the state-of-the-art precipitation nowcasting algorithm (ROVER; Woo and Wong, 2017). To the best of our knowledge, no research has been done in the SM prediction or other surface hydrology variables using ConvLSTM at the time of writing this article. In this study, we will perform analyses that are related to Fang et al., (2017), in the sense that we will predict SM values using hydrometeorological forcings. Our main sources of dynamic data input (predictors) are the High-Resolution Rapid Refresh (HRRR; Weygandt et al., 2009) and the Multi-Radar/Multi-Sensor radar rainfall product (MRMS; Zhang et al., 2016). Unlinke Fang et al., (2017) who used all outputs and forcings of the North American Land Data Assimilation System phase II (NLDAS-2; Xia et al., 2015) as predictors to their LSTM models, we selected a relatively smaller set of variables from HRRR in addition to MRMS rainfall as predictors to our models. This is done to test the effect of each of the predictors on the performance of the models. The reference SM product in this article is the output from the National Water Model (NWM) Noah-MP Land Surface Model (LSM) (Niu et al., 2011). It is important to note that the models can be trained to predict other sources of gridded SM data. The reason behind this choice is the availability of a long record of simulation outputs that are available on the National Water Center’s (NWC) Amazon Web Service (AWS) portal (https://registry.opendata.aws/nwm-archive/). The record spans from the year 1993 through the end of the year 2017 at the time of writing this article. We performed additional analyses to evaluate the effect of adding more predictors and the prolongation of the retrospective sequence of the LSTM component on the performance of the models. More importantly, we compared the performance of the ConvLSTM to that of CNN to assess the added benefit of incorporating the temporal aspect of the ConvLSTM models. As will be described later in this article, the data used to derive the NWM is different (reanalysis forcing data) from those used in the training process of DL models in this study. This adds to the credibility of the analysis by showing that the DL models can be generalized to predict SM from other sources.

Moreover, the analysis presented in this article included varying the number of predictors, and the length of the input sequences, to provide specific insight into the behavior of the ConvLSTM models for SM prediction. It is consequential to note that the number of possible combinations of predictors that can be used in DL models are overwhelmingly large and can come from various sources. In this study, we do not specifically focus on optimizing the combination of predictors, neither do we focus on finding the optimal length of the time series of past observations for the ConvLSTM models. We rather perform a pilot study to investigate the overall performance of ConvLSTM in hydrologic modeling and how it is affected by a limited set of predictors and sequence lengths. Another interesting aspect of this study is the ability of the ConvLSTM models to perform predictions in-between the discrete observations of a given SM product. This can be useful in the case of attempting to predict estimates of a product such as SMAP SM which is limited to the overpasses of the satellite (up to a few days). This is possible because the ConvLSTM models use independent predictors, thus, predictions can be made with the same temporal frequency of these predictors (hourly in our case). This process will be described in more detail later in this article.

In this study, we use open source software tools and free cloud computing services and storage resources without the need for a privately owned Graphical Processing Unit (GPU).

To summarize, the specific objectives of the current study are as follows:

1. Evaluate the performance of the ConvLSTM algorithm in predicting SM and compare its performance to the commonly used CNN algorithm.
2. Investigate the sensitivity of the ConvLSTM based models to the variability in predictor type and count.
3. Investigate the sensitivity of the ConvLSTM based models with respect to the length of the time series record (LSTM component) used in the learning and prediction process.
4. Demonstrate the model’s capability in producing predictions with a higher temporal frequency compared to the original frequency of the predicted variable (data gap-filling).

The remainder of this article is organized as follows. Section *Study Area and Datasets* describes the study area and the datasets used in the analysis. Section *Methods* describes the methods and how the data have been processed. Section *Experimental Setup* describes the experimental setup. Section *Results* presents the results and discussion of the analysis. Finally, conclusions are presented in Section *Discussion*.

## Study Area and Datasets

### Study Area and Period

**FIGURE 1:**
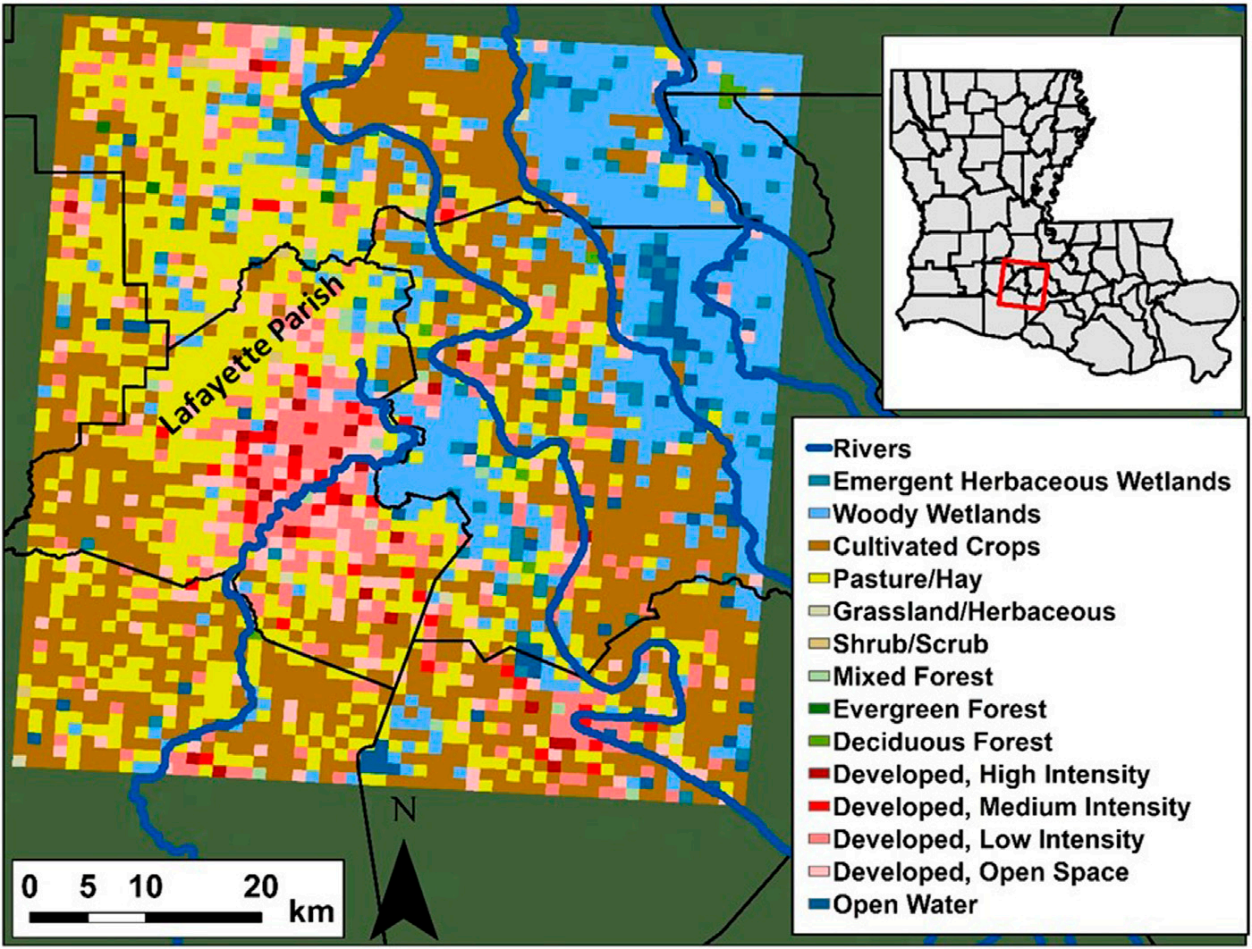
The study area is located in south Louisiana (red box in the top right). The left side of the figure depicts the different Land Use/Land Cover categories in the study area. The city of Lafayette is the developed area (shades of red) located in Lafayette Parish (mostly developed with different capacities).

The study area is located in south Louisiana, in the United States (Figure 1). It covers a domain that contains Lafayette parish (county) and its surrounding. Lafayette parish is fairly developed with a relatively dense population and has been frequently impacted by floods resulting from extreme rainfall events (Sharif et al., 2020). Additionally, the area lies in a very flat region due to its proximity to the Gulf of Mexico, this allows the water to stagnate on the ground and raise the water levels in the stream channels for elongated periods. Naturally, SM plays an immediate role in determining whether flooding will occur following a strong rainfall event or not based on the available soil’s ability to absorb more water. Lafayette parish is bordered by large wetland areas to the east (Figure 1) and a significant portion of it is occupied by the city of Lafayette which is mostly developed causing low soil capacity for water storage. The Vermilion River, which passes through the city of Lafayette, experiences complex hydrodynamic regimes during extreme rainfall events and frequently floods parts of the city of Lafayette and Lafayette parish. This is due to its flat slope (e.g., backwater flow) as well as the tidal flow patterns downstream of the river caused by the Gulf of Mexico. The top right portion of the study area is partially occupied by the Teche River basin which is a flood plain area that consists mainly of wetlands and open water bodies. The total area of the study domain is 3,575 km^2^ (65 km height × 55 km width). We gridded the domain at a 1 km × 1 km resolution to match the data inputs and outputs to the SM estimates obtained from the NWM. We also used the same projection parameters of the NWM’s LSM geogrid. The main Land Use/Land Cover categories obtained from the National Land Cover Dataset (NLCD; Homer et al., 2015 and Yang et al., 2018) in the study area are Woody wetlands (19.9%), Pasture/hay (25.3%), Cultivated Crops (32.4%), and various developed categories with different capacities (15.8%), all shown in Figure 1. The total population of Lafayette parish is 244,390 (https://www.census.gov/quickfacts/lafayetteparishlouisiana) which is mainly concentrated in the city of Lafayette. Our study period extends from August of 2016 through the end of the year 2017. In August of 2016, an extreme rainfall event resulted in rainfall depths of around 20–30 inches in some parts of the state with over 20 parishes including Lafayette parish declaring a state of emergency. This continuous period of hourly observations consists of 4,124 h. Combined with the number of pixels (65 × 55), this results in a total number of 14,743,300 data points per input variable. This number will be multiplied by up to 7 times (number of predictors) as discussed later in the article.

### The National Water Model Soil Moisture Data

We obtained the reference SM data from the NWM archive located and described in (https://registry.opendata.aws/nwm-archive/). The archive contains NWM reanalysis (retrospective) runs during 1993–2017. The NWM produces multiple outputs from its different components (e.g., LSM, and surface and channel routing components). The variable of interest (SM) in this study is an output of the LSM component of the NWM. Unlike the operational NWM runs (https://water.noaa.gov/about/nwm) that have an hourly frequency, the output frequency of the archived NWM retrospective runs is 3 hourly in the case of the LSM component. This is because the main purpose of this archive is to provide historical context to the current NWM outputs and intended to be used for frequency analysis and for algorithm training applications similar to what is performed in the current study. Volumetric soil moisture, the dimensionless ratio of water volume (m^3^) to soil volume (m^3^) (m^3^ m^−3^) is available on a 1 km × 1 km grid for the entire United States. We used this SM grid as a designated geographical setup for all other variables used in this study. Therefore, all other variables have been scaled to the NWM 1 km × 1 km grid. We subsetted (extracted) the NWM SM over our domain and stored the hourly grids in Tagged Image File Format (TIFF) using the Geospatial Data Abstraction Library (GDAL; GDAL/OGR Contributors, 2020) library of the Python programming language.

### High-Resolution Rapid Refresh Data Assimilation and Forecast Modeling System

The first dataset used as a predictor in this study’s DL models is obtained from HRRR model outputs (Weygandt et al., 2009). HRRR is a real-time atmospheric model operated by the National Oceanic and Atmospheric Administration (NOAA) covering the entire United States. The model has spatial and temporal resolutions of 3 km a 1-h respectively. HRRR is a convective-allowing, cloud-resolving atmospheric model that utilizes 3 km radar data assimilation every 15 min over hourly periods. In our study, we used HRRRv2 which was implemented at the National Center for Environmental Prediction (NCEP) from August 2016 through July 2018. A data HRRR data archive is provided by Blaylock et al., (2017), which was the source of HRRR data in this study. The total number of variables that the HRRR model produces is 132 variables. The variables used in this study are incoming longwave and shortwave fluxes, storm surface runoff, baseflow-groundwater runoff, and moisture availability.

### The Multi-Radar/Multi-Sensor Radar Rainfall Product

MRMS is a radar rainfall product that is gauge corrected in near real-time with spatial and temporal resolutions of 0.01 degrees (approximately 1 km × 1 km) and 1-h, respectively. The National Severe Storm Laboratory (NSSL) implements this product. MRMS has been extensively evaluated and validated for various applications (e.g., Zhang et al., 2016; ElSaadani et al., 2018; Sharif et al., 2020) and is available since September 2015. The product is archived by Iowa State University’s Iowa Environmental Mesonet (IEM) in GRIB format and is available at (https://mesonet.agron.iastate.edu/archive/). MRMS is an excellent source of continuous spatiotemporal rainfall observations over the entire United States and is widely used by the hydrologic community (Zhang et al. 2016; Sharif et al., 2020). In this study, rainfall observations are an irreplaceable source of information to train the DL models due to the direct relationships between rainfall and SM (Norbiato et al., 2008; Fang et al., 2017). Unlike some of the HRRR variables that weren’t used to train the DL models, rainfall observations are always included as a predictor for all of our models.

### National Land Cover Dataset

Another important input that we used to train the DL models is NLCD Land use/Land cover (LULC) dataset (Homer et al., 2015). The dataset covers the entire United States with a 30 m × 30 m resolution. We aggregated LULC data to 1 km × 1 km resolution to be consistent with other model inputs. As with rainfall observations, LULC data is crucial for the model learning process and is used as a static predictor for all our DL models. A breakdown of the available LULC classes over the study area is depicted in Figure 1. We used the latest available version of NLCD data at the time of this study (the year 2016) which is also consistent with the study period.

## Methods

### Data Representation

Although the NWM SM has a three-hourly resolution, the temporal resolution of the predictors (i.e., HRRR and MRMS) remains hourly. This is because based on our setup, we use this information between SM grids (NWM output) to enhance the prediction using the LSTM component of the ConvLSTM neural network. The input datasets are represented as a temporal sequence of hourly records, and each record is viewed as a three-dimensional grid or image (width = 55, height = 65, and depth = the number of predictors).

### Convolutional Neural Network

The best deep neural network architecture that can be used when dealing with images (spatial data) is Convolutional Neural Networks (CNN; LeCun et al., 2015). Hierarchically, CNNs can learn spatial patterns in the input data very effectively. In data sources like images, the neighboring pixels have a strong correlation and they can contribute together to form a spatial pattern in the data while remote pixels do not affect those patterns. Therefore, when learning the local patterns from a certain group of pixels in a specific area of an image, using fully connected layers of neurons won’t be very effective. CNN architecture typically comprises three different types of layers: alternating convolutional and pooling layers followed by a fully connected layer. A Convolutional layer consists of filters (kernels). These filters slide over (convolve) with the input image. The region with which the filter is convolved in the input image is defined by a configurable parameter (stride). The output of a convolutional layer is feature maps where the number of feature maps equals the number of the applied filters. The pooling layer performs a network down-sampling by lowering the dimension of the convolution layer output (feature maps). Ultimately, the fully connected layer combines all activations from the preceding layer and generates the output based on the objective of the neural network-based system.

### ConvLSTM

Considering that we have a dataset in the form of sequences or time series, it is reasonable to propose a system that uses a recurrent neural network (RNN) that can capture and identify the temporal patterns in the data. A recurrent neural network is a type of neural network that can maintain a state between various inputs. This state (memory) stores information about what the network has learned from the sequential data inputs. Long short-term memory (LSTM), a specific RNN architecture, introduced the concept of memory cells (units) with controlling gates to help maintain gradients while being backpropagated during network training and retain long-term temporal dependencies between inputs. An LSTM cell has three gates which are named the input gate, the output gate, and the forget gate. These gates regulate the information flow into and out of the LSTM cell by either storing or forgetting the previous state and passing or discarding the current state.

In our study, since we are working with a sequence of three-dimensional spatial data, a better approach is to benefit from the combination of LSTM and CNNs and deploy a convolutional LSTM (ConvLSTM; Xingjian et al., 2015) network. ConvLSTM networks consist of recurrent layers, just like the LSTM, but in each gate, a fully connected layer is replaced with a convolution layer and so, the internal matrix multiplications in a ConvLSTM cell are exchanged with convolution operations to help capture the underlying spatial features besides temporal features in our three-dimensional data.

### Proposed Systems Architectures

In this study, we are predicting the soil moisture (SM) every three hours based on hourly-recorded input data consisting of sequences of records. Each record consists of a three-dimensional image of the size 65 × 55 × V. The number of variables (channels or predictors) under analysis defines the third dimension (V) of the data. We have done our experiments using two different values of V: five, and seven.

The proposed system consists of four ConvLSTM layers stacked on each other. The output of the last layer is forwarded to one convolutional layer to output the final prediction. Two different experimental setups have been conducted for generating and testing different input sequences in the prediction process. In the first one, an input sequence consists of three or five consecutive hours of recorded data that includes data recorded at the same hour as the desired output (We name this experiment as Inclusive). In the other experiment, the input sequence consists of three or five consecutive hours of recorded input that precedes or exclude the hour of the desired output (We name this experiment as Exclusive). It is important to note that the frequency of soil moisture data is three hourly with the simulated value described as an instantaneous value during the first hour of the three hourly interval. Hence, the exclusive/inclusive setup is intended to test how much does the rainfall occurring during the first hour of the three hour period has a significant effect on the soil moisture value. For more details on the NWM soil moisture calculation, readers are advised to review the NWM user manual (https://water.noaa.gov/about/nwm).

**FIGURE 2:**
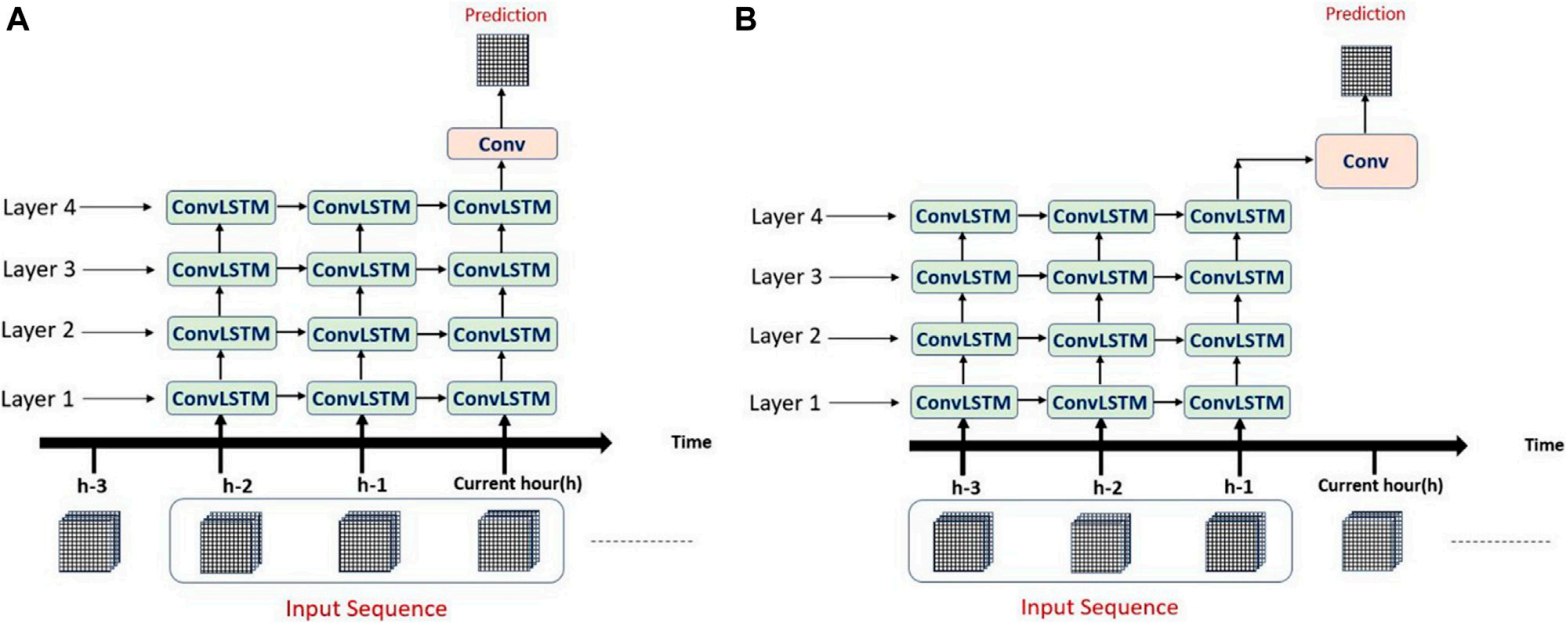
Unfolded representation of the ConvLSTM. **(A)** A single ConvLSTM sequence formation for an inclusive setup with a sequence length of three hours. **(B)** A single ConvLSTM sequence formation for an exclusive setup with a sequence length of three hours.

Figure 2A shows the first experimental setup in which a prediction at the current hour “*h*” is calculated based on the sequence inputs starting two hours before the current hour and ends including the input at the current hour “*h*”. This setup is called inclusive setup with sequence length 3. While Figure 2B shows the second experimental setup in which a prediction at the current hour “*h*” is calculated based on the sequence inputs starting three hours before the current hour and ends including the input at the previous hour “*h−1*”. Note that both figures show an unfolded representation of the ConvLSTM over the specified hours.

After using the grid search technique for hyperparameters tuning and evaluating a large number of ConvLSTM models with various configurations and based on the empirical results, we found that the best model should be set up as follows: The number of filters in each ConvLSTM layer is 64, 64, 50, and 32 respectively. A kernel size of 3 × 3 and the default hyperbolic tangent “*tanh*” activation function is used in each layer. To keep the same height and width at 65 and 55, all layers are configured using the same padding technique.

The final layer which is a convolutional layer has only one filter and the kernel size is 1 × 1 and uses the same padding technique. The activation function used in this layer is the sigmoid function to generate an output in the range 0–1.

### Model Tuning

Due to the nature of our prediction problem and the anticipated complex structure and the large number of parameters of the ConvLSTM model, considerable attention has been paid to avoiding the overfitting problem. Several regularization strategies can be used for reducing or preventing overfitting and helping the DL models to generalize when tested on unseen data. To speed up training and ensure high performance and stability of our model while removing the need for using typical regularization methods such as dropout, our model applies the batch normalization method Ioffe and Szegedy, 2015 between every two contiguous ConvLSTM layers. Different optimizers have been tested. Consequently, RMSprop (Tieleman and Hinton, 2012) was selected based on the model performance. The output prediction is in the range 0–1 so we were able to use the binary cross-entropy as the loss function as it outperformed the mean squared error loss function.

## Experimental Setup

The ConvLSTM model is implemented in python using Keras library with Tensorflow as a backend. We used the free online Google Colab service which provided us with free access to a Tesla K80 GPU. In this study, we produced a total of 12 DL models, four of them are pure CNN models and the rest are ConvLSTM models. The four CNN models vary based on two combinations of predictors, which we chose as examples to test the sensitivity of the models to the variation in predictors. It is important to note that MRMS rainfall and NLCD LULC were used in all models. We also performed exclusive CNN runs that utilized the values of the two combination of variables from hour “*h−1*” instead of the current hour “*h*” (Figure 2). The two combinations of variables beside rainfall and LULC are as follows:

1. Downward longwave radiation flux, downward short-wave radiation flux, and moisture availability. We refer to models utilizing this combination as the three-variables model for the remainder of this article.
2. Downward longwave radiation flux, downward short-wave radiation flux, moisture availability, storm surface runoff, and baseflow-groundwater runoff. We refer to models utilizing this combination as five-variables models.

In addition to the same predictor combinations used for the CNN models, we used different sequence lengths (3 h and 5 h) and different setups (i.e., inclusive and exclusive) for the ConvLSTM models. Furthermore, we picked one of the top-performing ConvLSTM models and applied to it the inter-observation technique to test the model’s capability in producing hourly SM predictions. Table 1 summarizes the three hourly DL models produced in this study.

**TABLE 1:** Summary of model combinations used in the study. The number of models within each category is shown in the parentheses. Categories are based on the number of additional variables included (in addition to MRMS rainfall and NLCD LULC).

Model category (count)	Additional predictors	Sequence length (hours)	Model setup
CNN 3 variables (two models)	Downward longwave radiation flux, downward short-wave radiation flux, and moisture availability	N/A	Inclusive or exclusive
CNN 5 variables (two models)	Downward longwave radiation flux, downward short-wave radiation flux, moisture availability, storm surface runoff, and baseflow-groundwater runoff	N/A	Inclusive or exclusive
ConvLSTM 3 variables (four models)	Same as CNN 3 variables	3 or 5	Inclusive or exclusive
ConvLSTM 5 variables (four models)	Same as CNN 5 variables	3 or 5	Inclusive or exclusive

### Data Preprocessing for Three Hourly Predictions (Original Soil Moisture Product Temporal Resolution)

As a preprocessing step, per each channel, min-max normalization to the 0–1 range is applied on the dataset inputs. The purpose of this min-max normalization is to ensure that all data points (pixels) are scaled similarly such that all pixels act as equally significant features. The soil moisture output (available every three hours) is min-max normalized into the range 0–1 as well. The whole dataset is then converted into 4,142 sequences of three or five hours based on the model sequence (e.g., Figure 2).

### Data Preprocessing for Hourly Prediction (in Between Observations)

**FIGURE 3:**
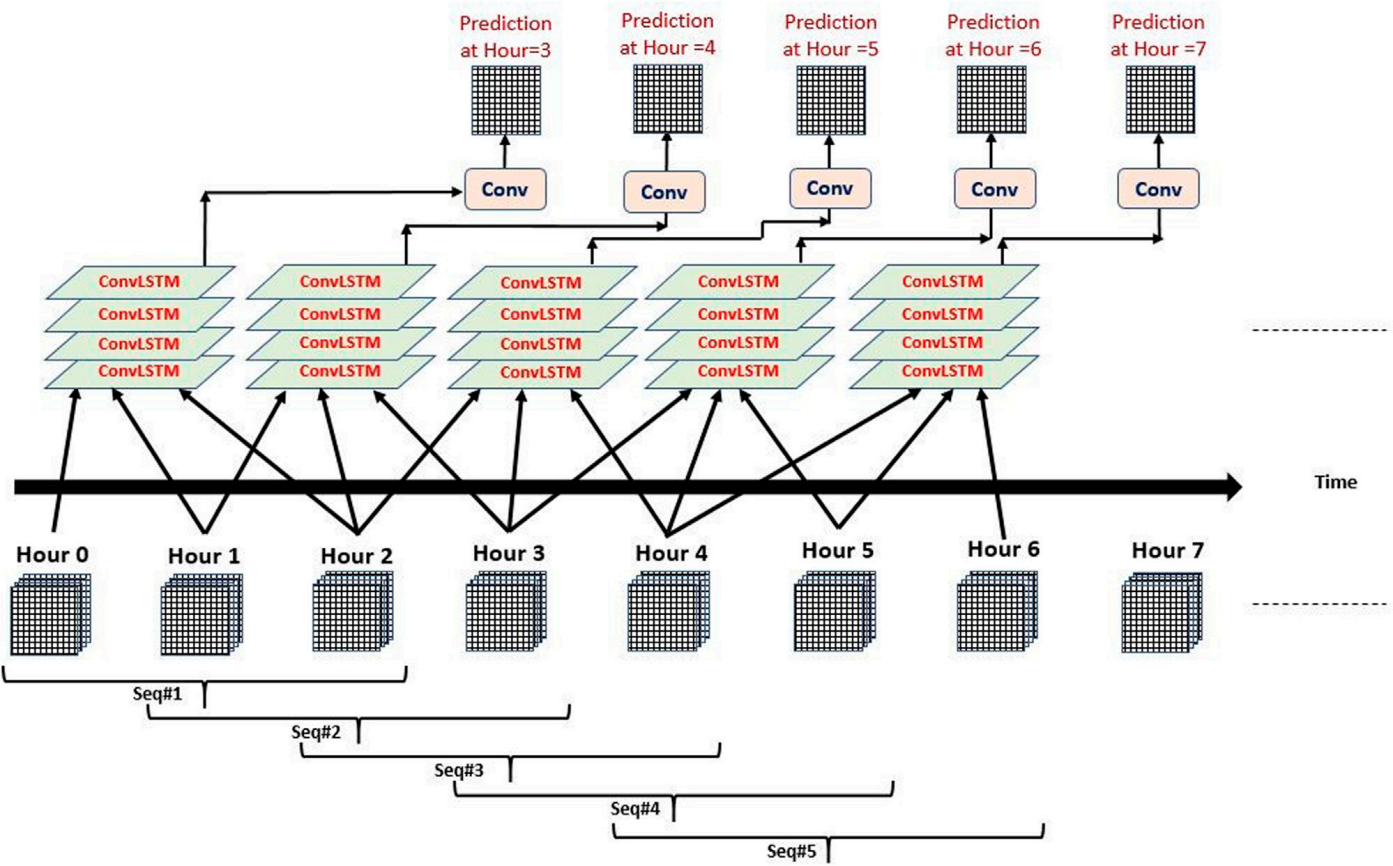
Multiple ConvLSTM input sequences output prediction every one hour “Exclusive” Sequence length is three hours.

To test the data gap filling prediction capabilities of our models, another testing setup is used to predict SM every hour. As an example, Figure 3 shows how multiple exclusive three hourly sequences are constructed to predict the soil moisture every hour. Inputs at hours 0, 1, and 2 are used to predict the output at hour = 3, and inputs at hours 1, 2, and 3 are used to predict the output at the next hour which is hour = 4, etc. Similarly, the setup can be applied to the 5 hourly sequences as well.

### Training, Validation, Testing Set Preparation

For training and testing the ConvLSTM model, the dataset is divided into two parts: a training set that contains three or five-hour sequences starting from August 1st, 2016 till July 31st, 2017 and a testing set that contains three or five-hour sequences starting from August 1st, 2017 till December 31st, 2017. That divides the dataset into a 12 months training set and a five months testing set. Moreover, the training set is randomly divided into a 90% training set which is used to train the model and the remaining 10% is used as a validation set for tuning the model hyper-parameters while training. All model variations were trained using backpropagation for a maximum allowed number of epochs equal to 50 and a batch size equal to 5. The number of epochs is selected empirically when the error and validation loss stops improving (early stopping). The average number of epochs that satisfied this criterion is approximately 30 epochs.

## Results

A total number of 12 DL models were created in this study, four of them are CNN-only models and the rest are ConvLSTM models. As described in the methods section, if the model uses the predictor data from a sequence ending in the current time step (i.e., lag 0 h) we call it an inclusive model. If the model uses the information of a sequence ending one hour before the current time step (i.e., lag 1 h) we call it an exclusive model. There are no LSTM sequences for CNN-only models and we only have four CNN models corresponding to the two combinations of variables in an inclusive and exclusive modes. In the case of ConvLSTM models, we tried two types of sequences that can utilize information from three or five-time steps. Thus, the total number of ConvLSTM models is eight (2 variable combinations, 2 time sequences, exclusive or inclusive).

### Overall Training and Testing Performance of Deep Learning Models

**FIGURE 4:**
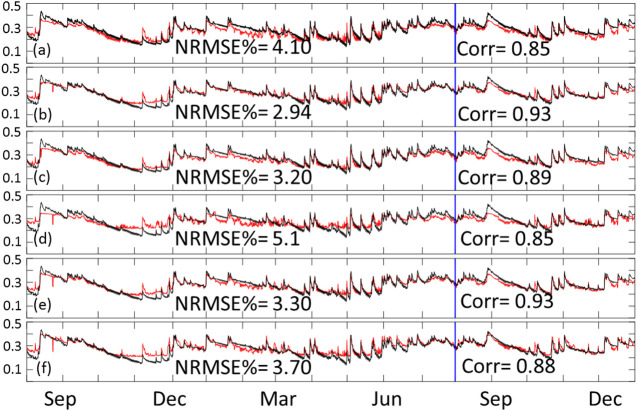
Time series of training and testing domain average for inclusive models. The red line represents the predicted data while the black line represents our reference SM product. Panel **(A)** shows the times series of CNN with 3 variable, **(B)** ConvLSTM sequence 3 with 3 variables, **(C)** ConvLSTM sequence 5 with 3 variables, **(D)** CNN with 5 variables, **(E)** ConvLSTM sequence 3 with 5 variables, and **(F)** ConvLSTM sequence 5 with 5 variables. Training data is from August 2016 through July 2017, while testing data is from August 2017 through December of 2017.

We started the analysis by looking at the overall performance of the models represented by the time series of the spatial average SM value calculated for the entire domain (65 × 55). This is done by obtaining the average of the domain values for each time step and presenting them as time series. Figure 4 shows the performance of the six inclusive models. In order to assess the performance of the simulation results (red lines) compared to the reference SM values (black lines), we calculated the normalized root mean square error as a percentage of the mean reference SM, as well as the correlation coefficient between the two time series. As seen in the figure, the least performing models are the CNN-only models shown in panels (A) CNN 3 variable, and (D) CNN 5 variables. Meaning, the ConvLSTM models consistently outperformed the CNN-only models. The figure also shows that the three variables models (panels a through C) outperformed the five variables models (D through E). This suggests that the two added variables (surface and subsurface runoff) did not help improve the model training process. Moreover, the ConvLSTM models with a sequence length of 3 outperformed those with a sequence length of 5. This suggests that including information beyond 3 h in the past is not useful for the training process.

**FIGURE 5:**
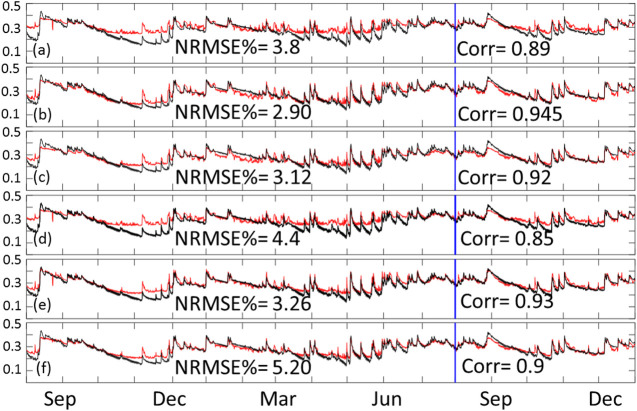
Time series of training and testing domain average for exclusive models. The red line represents the predicted data while the black line represents our reference SM product. Panel **(A)** shows the times series of CNN with 3 variable, **(B)** ConvLSTM sequence 3 with 3 variables, **(C)** ConvLSTM sequence 5 with 3 variables, **(D)** CNN with 5 variables, **(E)** ConvLSTM sequence 3 with 5 variables, and **(F)** ConvLSTM sequence 5 with 5 variables. Training data is from August 2016 through July 2017, while testing data is from August 2017 through December of 2017.

Figure 5 is the same as Figure 4 but for the exclusive configuration. As seen in the figure, the exclusive configuration models performed slightly better compared to their corresponding models in the inclusive configuration. This suggests that the information from the current time step is less relevant to the training process compared to that of the previous time steps. Given that the SM provided by the NWM represents instantaneous values during the first hour of a 3 h time step (as described in the NWM manual), this might explain why the rainfall, runoff, and moisture availability information at the end of that first hour did not contribute to the SM value (due to the time needed for infiltration). The figure also shows relative performance between the exclusive models that is consistent with what is observed in Figure 4 (i.e., 3 variables outperformed 5 variables, and sequence 3 slightly outperformed sequence 5).

### Spatial Performance

**FIGURE 6:**
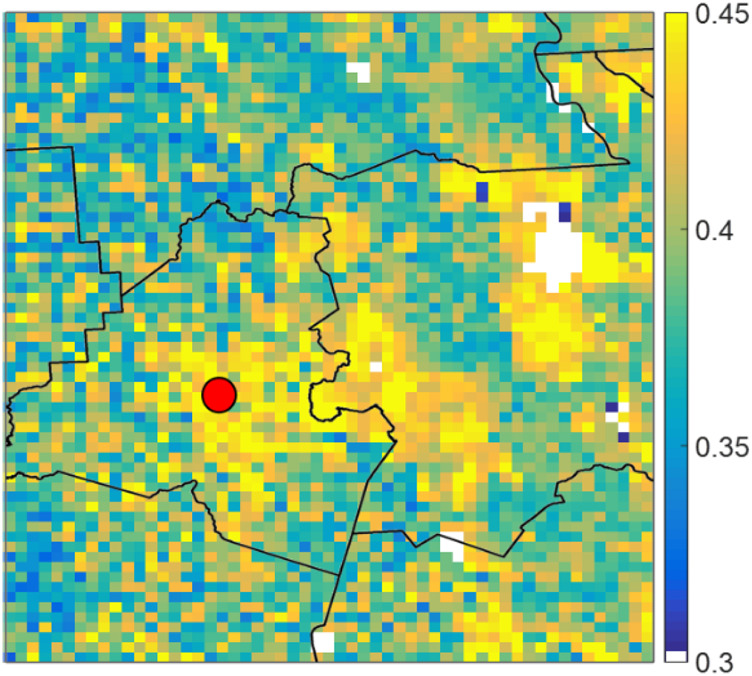
Average spatial SM field for the study area. White clusters depict water bodies in the study area. Black polylines depict parish boundaries, while the red dot represents the location of the city of Lafayette.

In this subsection, we investigate the models’ performance in more detail where we focus on the performance in a spatial context. This is done by calculating the skill score percent normalized root mean squared error (NRMSE%) and the correlation coefficient using the time series of each pixel rather than the domain average. Each pixel has its own two time series of reference and predicted SM, similar to what is shown in the previous section. We then calculated the skill scores on a pixel-by-pixel basis and plotted them spatially. To calculate the (NRMSE %), we normalized the RMSE as a percentage of the mean of the reference SM. Thus, we start by showing the mean SM field of our domain in Figure 6. The white clusters in the figure depict water body areas where SM is not to be measured (also shown in Figure 1). The highest soil moisture clusters are located in the wetland areas in the Teche region and immediately east of Lafayette Parish. It is important to note that volumetric SM depends on soil storage capacity, thus the values at highly developed areas have high values (e.g., the city of Lafayette). In contrast to natural LULC areas, which vary in SM over time, the SM content for developed areas is constantly high due to low soil storage capacity (almost impervious).

**FIGURE 7:**
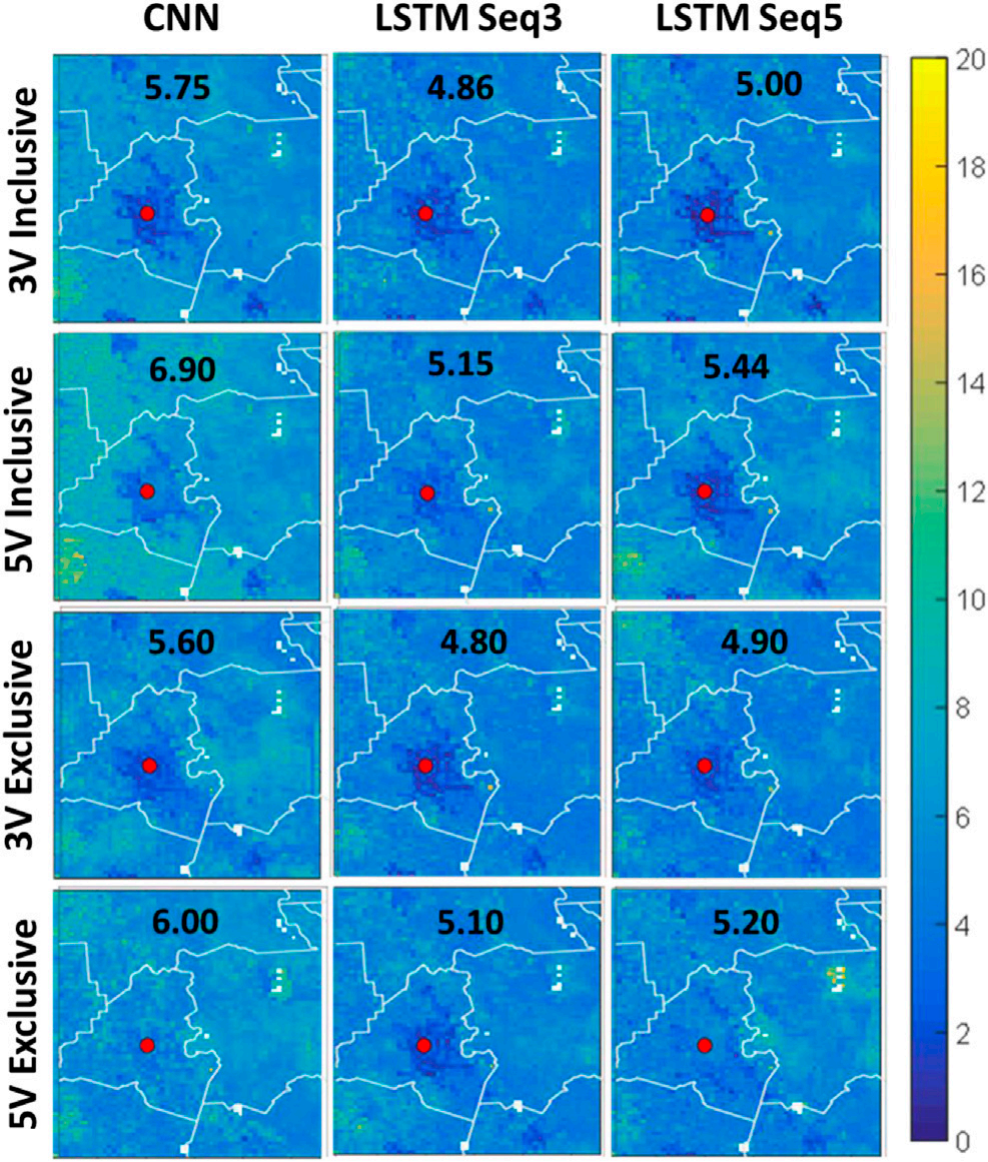
Spatial NRMSE% fields for the study area. The top two rows represent 3 variable and 5 variable models in inclusive configuration, while the last two rows are the same but for exclusive configuration. The first column represents CNN-only models, while the second represents ConvLSTM with sequence length of 3, and finally the last column is for ConvLSTM with sequence length 5.

Figure 7 shows the spatial NRMSE% fields for the study area. A common feature in all subplots of this figure is that NRMSE% is higher in some clusters that correspond to cultivated crops and pasture/hay LULC areas. This is due to the low-mean SM values over these areas (Figure 6); this magnifies the effect of errors when compared to the mean values. On the other hand, NRMSE% is always very low over the city of Lafayette (red dot), which is the opposite of the previous case since the SM values are always high in this area (because urban LULC has little water storage capacity) easing the effect of errors when compared to the mean SM value. As seen in Figure 7, although the best performing models from the previous subsections are generally the same; their performance varies within the domain. Moreover, the variables added in the five-variables models’ configuration did not add to the quality of the models making the three variables models’ overall spatial performance better. In addition, the exclusive configuration models consistently outperformed the inclusive configuration models. Visually, the exclusive sequence 3 model with 3 variables is the best overall performance. Overall, most ConvLSTM models had errors consistently less than 10% over most of the domain.

**FIGURE 8:**
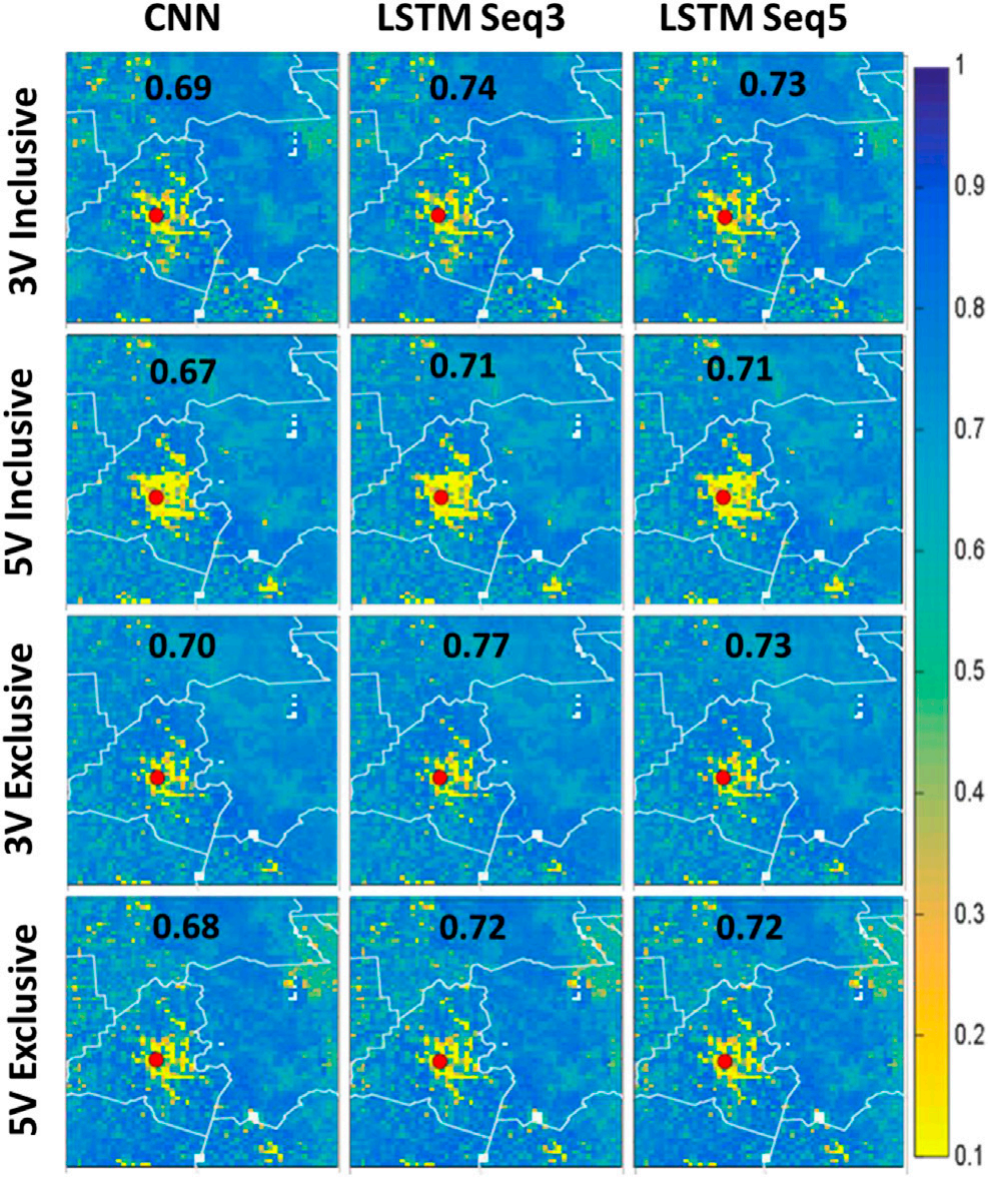
Spatial correlation fields for the study area. The top two rows represent the 3 variable and 5 variable models in inclusive configuration, while the last two rows are the same but for exclusive configuration. The first column represents CNN-only models, while the second represents ConvLSTM with sequence length of 3, and finally the last column is for ConvLSTM with sequence length 5.

Figure 8 shows the spatial correlation fields for the study area. The results shown in this figure are consistent with those presented in Figure 7. Nevertheless, despite the very low NRMSE% over the Lafayette area, the correlation is very low. This might be due to the variability of SM values in this area is low (always high volumetric SM as presented in the model). This is difficult for the models to replicate precisely given the high variability in SM everywhere else in the study area. Sequence 3 exclusive model with 3 was again the best overall performer.

**FIGURE 9:**
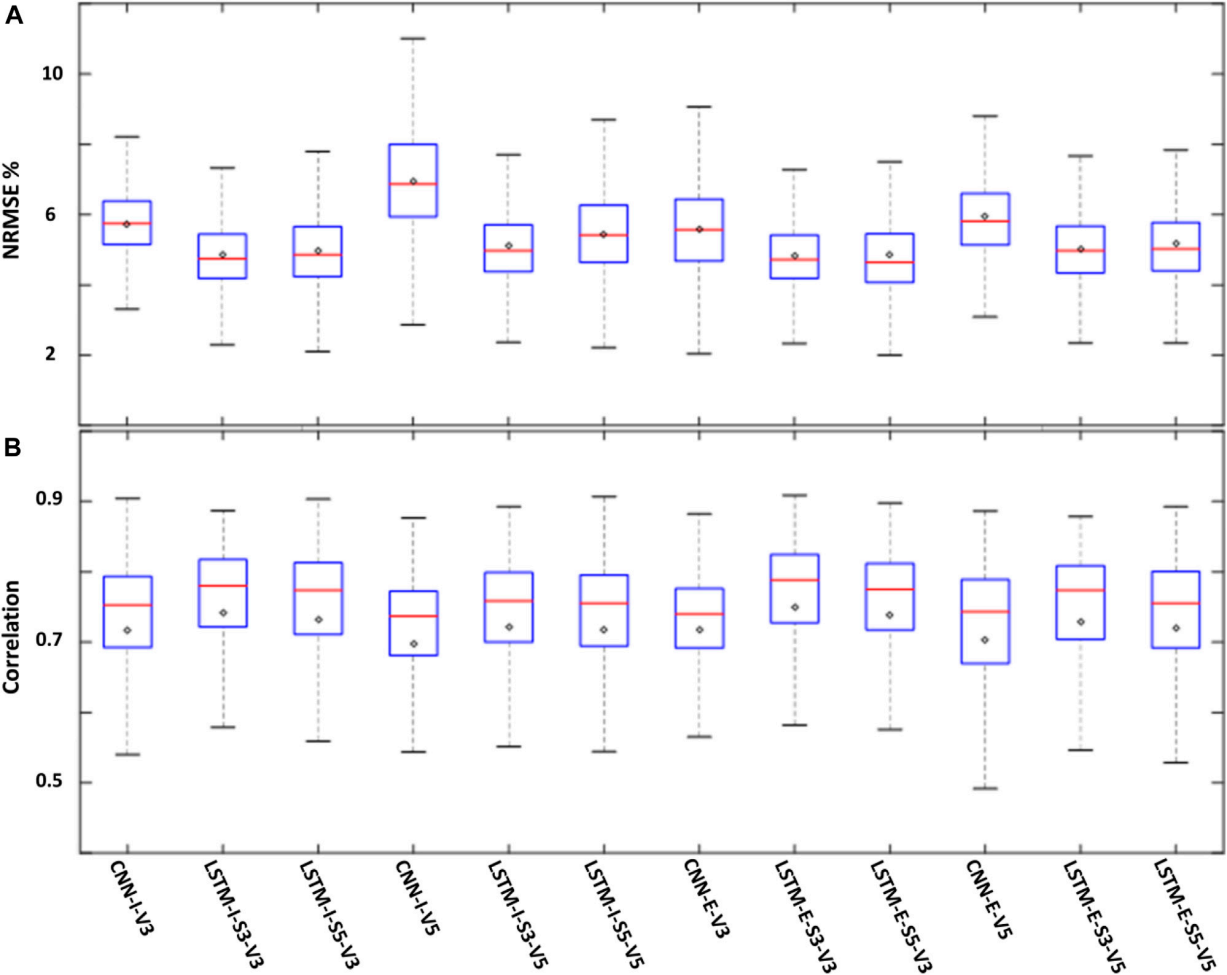
Box plots showing the NRMSE% **(A)** and correlation **(B)** for all models. The top edge of each box represents the 75th percentile while the bottom edge represents the 25th percentile. The red lines represent the median (50th percentile) and the black diamonds represent the mean value for each model.

To complement the visual investigation of the spatial plots in Figure 7, box plots of NRMSE% for all models are shown in Figure 9A. The figure suggests that inclusive models had higher variability in their pixel values which in turn affected their median (red line) and mean (black diamond) values. Overall, the ConvLSTM 3 variables models with sequence length 3 were the best performing models.

Figure 9B is similar to Figure 9A but for correlation values. All outperforming models had a good correlation with median values approaching 0.8. The mean values for these models are slightly less than the median values suggesting that the models had a significantly poor performance in certain pixels compared to the overall model performance.

### Effect of Land Use/Land Cover

**FIGURE 10:**
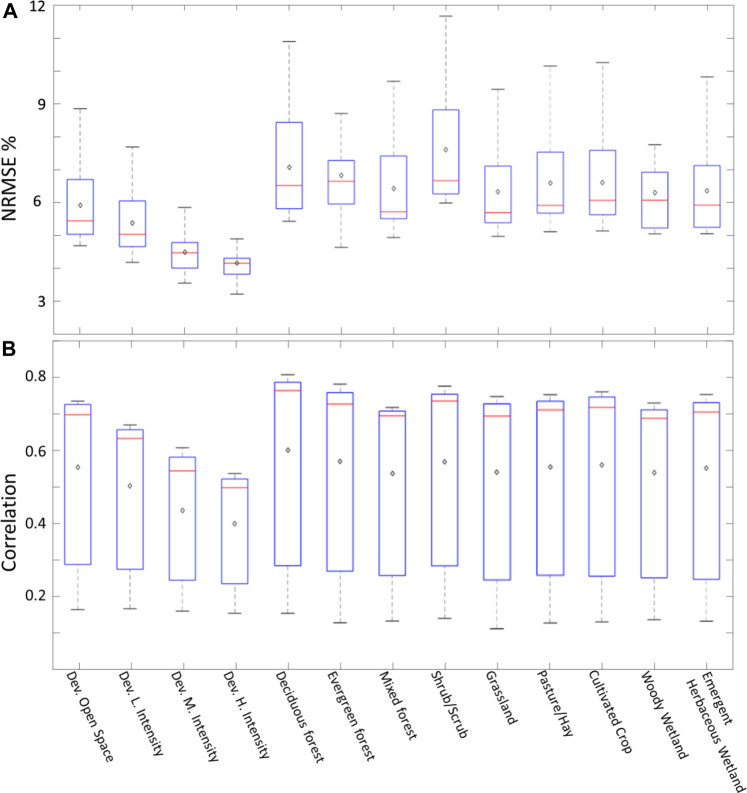
Box plots showing the NRMSE% **(A)** and correlation **(B)** for all models corresponding to each LULC. The top edge of each box represents the 75th percentile while the bottom edge represents the 25th percentile. The red lines represent the median (50th percentile) and the black diamonds represent the mean value for each LULC.

The performance of the models with regards to the corresponding LULC is examined by averaging the performance statistics (NRMSE% and correlation) of each model over a given LULC type. For example, the first box in Figure10A shows the distribution of the mean NRMSE% values obtained from each model for the developed open space LULC. As expected, the best NRMSE% performance corresponds to high intensity developed areas (Figure 7). The least performing LULC is the shrub/scrub areas and deciduous forest. All other LULC categories performed similarly. We produced the same analysis for the correlation values in Figure 10B. The least correlation was observed over high intensity developed areas similar to what is shown in Figure 10. Most of the other LULC categories performed similarly in terms of correlation.

### Data Gap Filling Capability

The capability of the DL models in predicting inter-observations is explored. As described earlier in this article, the frequency of the reference SM grids is 3-hourly. To predict the values every three hours, we generated several predictor sequences that end at (inclusive) or one hour prior to (exclusive) the desired prediction time step. Nevertheless, our models are not limited to predicting at the reference observations. It is of course important to produce predictions at the exact time steps of the reference SM to be able to validate the predictions. However, once the DL model predictive capabilities are validated, it can be used to predict at time steps whenever enough predictor sequences are available. Meaning, if a prediction is made at a certain time step, we do not need to wait for another 3 h to make another prediction, but rather predict after one hour only using the sequence of the previous 3 or 5 h of predictors. This can be explained easily using SMAP observations as an example. If the satellite overpass frequency is approximately every three days, we are not limited to making observations once every three days but rather make predictions based on the frequency of the used predictors in the middle of overpasses. This process is very similar to temporal downscaling.

**FIGURE 11:**
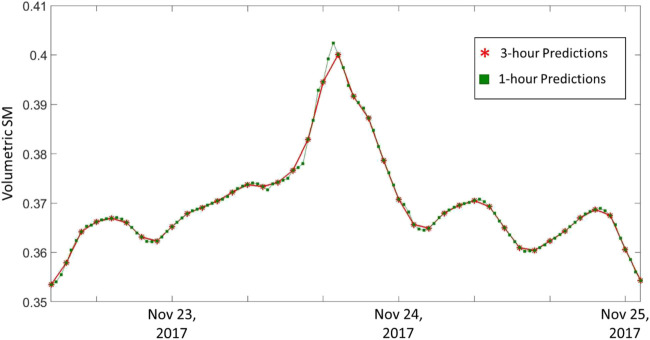
Time series of predicted 3-h spatial averaged SM (Red asterisks) vs 1-h spatial averaged (Green squares).

In order to illustrate this capability, we examine the model performance for the period between November 22, 2017, and November 25, 2017, using one of the top-performing models ConvLSTM sequence 3 with 3 variables in an inclusive setup (Figure 11). The red asterisks represent the predictions performed every 3 h while the green squares represent the hourly predictions. As expected, both hourly and 3 hourly predictions overlap at the times of reference observations. This is because we used the same model that was trained using the training period of observations, but fed it with additional predictor sequences in the prediction step. As illustrated in Figure 11, this allowed for a higher detail (temporal resolution) in the prediction time series especially during peak events where one would expect more activity in between the original time steps of three hours.

## Discussion

In this article, we developed DL models to predict SM over Lafayette parish and its surroundings in southwest Louisiana, United States. The area is covered mostly by open areas (cultivated crop, scrub/scrub, pasture/hay) and wetlands. The remainder of the study area is covered by developed areas with different intensities mostly within the city of Lafayette. The first set of models are CNN models that take spatial autocorrelation into account, and as such are well-suited to capture spatial patterns. The second type of model developed in this study, ConvLSTM models, combine the spatial capabilities of CNN models with the time series accounting capabilities of LSTM models. Our experimental setup was designed to show the added value of using the predictors’ sequence information from previous time steps in improving the models’ predictive capabilities. The work presented in this article shows that adding information from past observations can significantly improve the models’ predictive capabilities. In addition, we tested the value of adding more predictors on the models’ performance using up to 5 predictors in combination with rainfall and LULC. This article did not focus on finding the optimal combination of variables or sequence length but rather on the relative performance that adding additional information can have on the models. Our results show that ConvLSTM models can predict SM values spatially with mean and median pixel NRMSE% values close to 5% and maximum values below 10%. The models performed relatively better in terms of NRMSE% in areas where the mean reference SM is higher. Mean and median pixel correlation values of most of the ConvLSTM models were between 0.7 and 0.8. This indicates good agreement between the predicted and reference SM; and that they significantly outperformed CNN models. The NRMSE% and correlation coefficient values are much better while predicting the mean value of the entire domain with NRMSE% values as low as 2.9% and correlation values are above 0.9. Moisture availability defined as the ratio of actual to potential evapotranspiration added the most value to the models’ predictive abilities. LULC type had a significant effect on the performance of the models where areas with higher SM variability exhibiting higher error values and areas with consistently high SM values such as developed areas exhibiting lower error values. Besides, we were able to perform additional analysis to show the predictive ability of the DL models between observations. This is especially useful for applications such as filling of satellite observations in between overpasses. The models produced in this study can be scaled and applied to other SM products and geographical regions. Another interesting aspect of the study is that it has been conducted entirely using free open-source computational resources provided by Google Colab. The models are digitally stored in an HDF5 file format and can be easily loaded and trained using additional data when available. The codes used in this article can be obtained from the link are available to download freely (please check the article data sharing information).

## Conclusion

In this article, we presented a new approach for data-based SM prediction using ConvLSTM DL models. The models can be generalized and scaled to predict SM observations from other sources. The main reason we decided to use the SM output of the NWM is due to its long available record and its high resolution in space and time. As more accurate SM observations (e.g., those collected by SMAP) are available in the future, the models can be easily modified to be trained to predict them. The following conclusions are made based on the results of the study:

1. ConvLSTM models produced significantly improved SM prediction in comparison to CNN models indicating the significance of including past information in predicting the current state of SM.
2. Additional predictors such as storm surface runoff and baseflow-groundwater runoff (5 variables models) did not improve the performance of the models.
3. The past observation sequence length of 3 h allowed the ConvLSTM models to outperform CNN models significantly. Nevertheless, adding additional information from previous time steps (e.g., sequence length of 5 h) did not improve the performance of the models and in some cases caused deterioration of the models’ performance.
4. ConvLSTM models can replicate hydrologic models’ results with mean and median pixel-based NRMSE% around 5% and correlation coefficients around 0.8 using different sources of inputs that were not used to derive the hydrologic models. This is a good indicator that the models can be generalized to predict other sources of SM observations such as SMAP. The NRMES% and correlation for mean areal SM prediction over the study area of the best performing models were 2.9% and 0.91 percent respectively.
5. ConvLSTM models can predict SM value between the observations of our reference SM. This is an important feature that can allow the models to predicted SM observations of products like SMAP between satellite overpasses.

## Data Availability

The original contributions presented in the study are included in the article/Supplementary Material, further inquiries can be directed to the corresponding author. The codes used in the study are available at the following link https://gitfront.io/r/ahmedabdelhameed/bde79680b01cba747d5e15a1c126c637fff6d134/Convlstm-SM/
